# The potential dangers of not understanding COVID-19 public health restrictions in dementia: “It’s a groundhog day – every single day she does not understand why she can’t go out for a walk”

**DOI:** 10.1186/s12889-021-10815-8

**Published:** 2021-04-20

**Authors:** Clarissa Giebel, Kerry Hanna, Manoj Rajagopal, Aravind Komuravelli, Jacqueline Cannon, Justine Shenton, Ruth Eley, Anna Gaughan, Steve Callaghan, Hilary Tetlow, Stan Limbert, Rosie Whittington, Carol Rogers, Kym Ward, Lisa Shaw, Sarah Butchard, Mark Gabbay

**Affiliations:** 1 Department of Primary Care & Mental Health, University of Liverpool, Brownlow Street, Liverpool, L69 3GL UK; 2 NIHR ARC NWC, Liverpool, UK; 3 Lancashire and South Cumbria NHS Trust, Preston, UK; 4 North West Boroughs NHS Trust, Warrington, UK; 5 Lewy Body Society, Wigan, UK; 6 Sefton Older People’s Forum, Liverpool, UK; 7 Liverpool Dementia Action Alliance, Liverpool, UK; 8 Together In Dementia Everyday (TIDE), Liverpool, UK; 9 EQE Health, Liverpool, UK; 10 SURF Liverpool, Liverpool, UK; 11 Me2U Day Care Centre, Liverpool, UK; 12 National Museums Liverpool, Liverpool, UK; 13 The Brain Charity, Liverpool, UK; 14 Department of Modern Languages and Cultures, University of Liverpool, Liverpool, UK

## Abstract

**Background:**

Sudden public health restrictions can be difficult to comprehend for people with cognitive deficits. However, these are even more important for them to adhere to due to their increased levels of vulnerability, particularly to COVID-19. With a lack of previous evidence, we explored the understanding and changes in adherence to COVID-19 public health restrictions over time in people living with dementia (PLWD).

**Methods:**

Unpaid carers and PLWD were interviewed over the phone in April 2020, shortly after the nationwide UK lockdown, with a proportion followed up from 24th June to 10th July. Participants were recruited via social care and third sector organisations across the UK, and via social media.

**Findings:**

A total of 70 interviews (50 baseline, 20 follow-up) were completed with unpaid carers and PLWD. Five themes emerged: Confusion and limited comprehension; Frustration and burden; Putting oneself in danger; Adherence to restrictions in wider society; (Un) changed perceptions. Most carers reported limited to no understanding of the public health measures in PLWD, causing distress and frustration for both the carer and the PLWD. Due to the lack of understanding, some PLWD put themselves in dangerous situations without adhering to the restrictions. PLWD with cognitive capacity who participated understood the measures and adhered to these.

**Discussion:**

In light of the new second wave of the pandemic, public health measures need to be simpler for PLWD to avoid unwilful non-adherence. Society also needs to be more adaptive to the needs of people with cognitive disabilities more widely, as blanket rules cause distress to the lives of those affected by dementia.

**Supplementary Information:**

The online version contains supplementary material available at 10.1186/s12889-021-10815-8.

## Background

With over 50 million people living with dementia worldwide [1], it was expected for the COVID-19 pandemic to have a particular impact on the lives of people living with dementia (PWLD) and their carers [2]. Dementia manifests itself in different symptoms depending on the dementia subtype, affecting cognition, behaviour, language, and mobility [3–5], and together with other neurological conditions are the third most common cause of disability in Europe [6].

In the UK alone, over 850,000 people are living with dementia since the last statistics were published in 2014, although it is estimated that currently over 1 million people are living with dementia in the UK (Alzheimer’s Society, 2014). Public health measures have varied since the first UK lockdown on the 23rd of March and it is important to adapt public health measures to current circumstances. At the time of this study, the UK saw a nationwide lockdown from March to June, with restrictions slowly eased end of June and early July. Vulnerable people were still shielding until early August, which meant that older adults and those with underlying health conditions were suggested to not leave the house and avoid any social contact, including in supermarkets. This meant that some people were allowed to go outside and others were told to stay at home at all times if possible. These sudden changes and different types of public health measures can be confusing to remember for people with cognitive deficits, such as PLWD. In the majority of dementia subtypes, including Alzheimer’s disease dementia and Lewy Body dementia, retaining new information is severely impacted [7, 8]. This ability deteriorates further as the dementia progresses, so that people in the moderate and more advanced stages are likely to struggle to a much greater extent to understand the public health restrictions and retain their nuanced and changing nature.

Early evidence indicates that PLWD have deteriorated faster in the first few months of the global pandemic [9, 10]. Unpaid carers reported faster symptom deterioration of their relatives with dementia due to the sudden withdrawal of accessing social support services and socially interacting with people, and the lack of physical and cognitive stimulation due to being restricted to and isolated in the home environment. In light of recently introduced stricter public health measures, it is important for restrictions to be comprehensible to PLWD and for services to implement restrictions to the benefit of those living with dementia and caring for someone with dementia.

The aim of this study was to explore the level of understanding and adherence to COVID-19 public health restrictions in PLWD and how this might have changed from the beginning of the pandemic. Considering imminent further regional and national lockdowns in the UK for the foreseeable future, as well as globally with a second spike in virus cases reported, it is important to understand the level of comprehension and resulting adherence to these life-saving COVID-19 public health restrictions in PLWD, as one subgroup of the most vulnerable of our societies. Findings will have wider implications for people with cognitive deficits and how restrictions need to be communicated and possibly adjusted to support PLWD and those with cognitive deficits better during this ongoing pandemic.

## Methods

### Participants and recruitment

Unpaid carers and PLWD with mental capacity aged 18+ and residing in the UK were eligible to take part. Participants were recruited via convenience sampling in April 2020 via social support service and third sector organisations via email mail outs, newsletters, direct telephone recruitments, as well as wider via social media. Details of interested carers and participants were forwarded to the lead researcher, and interested carers and PLWD could contact the lead researcher directly. Given the urgency to conduct the research in a timely manner to collect data on the experiences at this point in time, participants were selected in order of approaching us/contact details provided to us.

We obtained ethical approval from the [blinded for review] prior to the study [Ref: 7626].

### Data collection

Participants took part in telephone interviews, which lasted between 20 to 60 min. Baseline interviews were conducted in April 2020, and follow-up interviews were conducted between the 24th of June to the 10th of July 2020. The interview topic guides for the baseline and follow-up interviews were developed jointly with carers and a PLWD as well as social support service organisations and clinicians. The development process involved talking about the different topics that needed to be captured and joint discussions of iterations of the topic guide, pulled together by the lead author. This ensured that the voices of people affected by dementia and those providing services for dementia were filtering in into the topic guides and the questions being asked. Both topic guides are attached in Additional file 1. Trained qualitative researchers with PhDs (CG, KH), both researchers at the University, and DClin Trainees conducted the telephone interviews. At the beginning of each interview (baseline and follow-up), verbal informed consent was taken which involved ensuring that participants knew what the study was about, had had opportunity to ask questions, and were aware of the anonymity and voluntary nature of study participation. Audio recordings were subsequently transcribed.

### Data analysis

All interviews were transcribed verbatim, and subsequently double-coded, manually, by the research team (CG, MG, KH, SB, JC, SC). This involved each transcript being coded by two research team members, to ensure that no information was missed and to strengthen the data analysis quality. Descriptive thematic analysis was undertaken on the 70 transcripts, following the six phases of analysis outlined by Braun & Clarke [11]. Emergent codes were first produced inductively by individual coders. The research team later met to develop the initial themes by discussing the emerged codes and ensure adequate representation of findings was achieved. The five final themes were agreed by all members of the team, including carers, a person living with dementia, third sector providers, clinicians, and academics.

## Results

A total of 50 unpaid carers (*n* = 42) and PLWD (*n* = 8) participated in the baseline interviews, with 20 unpaid carers (*n* = 16) and PLWD (*n* = 4) completing a follow-up interview 3–4 months later. The majority of the baseline participants were female (76%). Carers were mostly spousal carers (55%) and living with the PLWD. The dementia subtype of those cared for and PLWD participating ranged from Alzheimer’s disease dementia (43%) to vascular (16%) and Lewy body dementia (6%). For further details on the baseline demographics see Giebel et al. [10].

We identified five themes that emerged from the qualitative transcripts: (1) Confusion and limited comprehension; (2) Frustration and burden; (3) Putting oneself in danger; (4) Adherence to restrictions in wider society; (5) (Un) changed perceptions.

### THEME 1: confusion and limited comprehension

Many carers described how the PLWD they were caring for did not, or only to an extent understand the various public health restrictions. Some PLWD apparently only understood some restrictions, whilst continuously forgetting the remainder. Many PLWD however as reported by their carers did not comprehend the restrictions at all.

*“I mean you have to laugh it’s a ground hog day. Every day every single day she does not understand why she cannot go outside for a walk, every single day and we go through the same thing, well actually we laugh about it because there is a kernel in her that knows she is you now its devilment it’s a, she can’t quite remember why she can’t go out, she does know that she can’t but she’s going to push it.”*

*Female carer (daughter), Baseline ID42*

*“He doesn’t understand at all. We have to explain it to him every day why he can’t go out. He knows that he can’t go because he knows that he’s not going to his groups but he doesn’t understand why he’s not going and he doesn’t understand why he can’t go out. So every day he says to me because I speak to him on the phone every day he says to me I’m going to get my coat on and I’m going to go and catch the bus. Well no actually Dad you can’t do that, well why so then we have to you know try and explain it to him again because there’s lots of germs out there and we don’t want him to become ill.”Female Carer (Daughter), Baseline ID38*

*“He is still not understanding what it means by the lockdown, obviously he's got the TV on all the time, he's always been an avid news watcher but when I’m talking to him he's saying you know so when is, about going the bank to get his money. And I’m going no the banks, they're not open dad you can only get your cash if you want to get the cash machine and obviously he wouldn’t be able to go the cash machine on his own to get that cash out and I’m having to remind him about things like that. And then another day and I will have to say the same things over again. So it’s not staying there.”Female carer (daughter), Baseline ID40.*

Very few carers described the PLWD understanding the public health restrictions fully. Where the PLWD did so, they were compliant with them.

*“[PLWD] is understanding entirely why he can’t go out, he's not anxious about it all. I've offered to take him round the green in the chair, no not bothered whatsoever about going out because he understands why he's here and why I can’t go out very often.”Female carer (spouse), Baseline ID22*

*“it was my daughter’s birthday and he came down again, obviously we are just keeping that distance giving him her card and he was understanding it was sort of blow a kiss you know and he was sort of obviously laughing at that and catching the kiss and you know playing along with that, he knows, he seems to understand the need to still keep that distance.”Female carer (daughter), Baseline ID40.*

This was reflected in PLWD who participated themselves, all of which had mental capacity and thus were in the earlier stages of the condition. Where PLWD shared their experiences of living with the public health restrictions, they appeared to comprehend the measures and why these were in place. PLWD felt very discontent with the impact these restrictions had on their lives, such as not being able to see their grandchildren, as well as long-term concerns for their children’s and grandchildren’s well-being and job security.

*“it’s all very scary [ … ] keep so much space between each other you know I will be much happier when it’s gone.”Male PLWD, Baseline ID37*

*“I’m not worried about it for myself personally because I've never been worried about dying or anything like that but I was worried about my family and my friends. I've got a granddaughter who is 8 like you know so I worry about her and worry about my son and then worrying about the impact on their jobs.”Male PLWD, Baseline ID45*

*“My son with my grandchildren lives ‘round the corner, so when I’m doing my exercise for an hour a day, I always go for a walk and I can only wave to them and shout to them, so I’m missing out a lot, I feel as though I’m missing out a lot.”Male PLWD, Baseline ID46.*

### THEME 2: frustration and burden

#### Behavioural changes in PLWD

The inability to comprehend public health restrictions, such as suddenly not being allowed to go outside for a walk anymore, or being unable to attend previously enjoyed support groups or activities in the community, left a number of PLWD with changed behaviours, as reported by their unpaid carers. PLWD could become frustrated and agitated at these sudden impediments to their previously enjoyed routines

*“the third week she kind of understood that we weren’t allowed to go out and we could go for a walk and we couldn’t do much shopping and we couldn’t do all the usual things like we’d go out and have a cup of tea somewhere and all that. The switch flipped you know, she couldn’t understand why we wouldn’t take her to the day centre she wanted to go out she wanted to do her own thing, she wanted to go shopping, she wanted and we were sort of like the horrible people that were stopping her from doing that so it really affected her and we got to the stage where she was really angry”Female carer (daughter), Baseline ID28*

*“I think they feel very frustrated and a bit isolated and [PLWD] looks out the window and she sees who she considers old people walking around [Park] and she doesn’t understand why she can’t and that adds to her frustration and even though we explained to her that she’s in a very special category”Female carer (daughter), Baseline ID42*

*“He was getting very agitated with the kids we both needed the break I think but now the weathers nice it’s you know we, he can be in the garden and stuff.”Female carer (daughter), Baseline ID24.*

#### Increased carer stress

In turn, with PLWD becoming more agitated or frustrated, many unpaid carers reported becoming more stressed by having to manage these behavioural changes. Moreover, having to explain why PLWD were suddenly not allowed to go outside to their social groups or day care centres anymore, or enjoy outdoor hobbies such as going to the garden centre, left many carers additionally stressed. Carers described how they continuously, several times a day, had to explain the public health restrictions to the PLWD.

*“you tell him as we’re going on a walk Dad you’ve got to keep away and as soon as he see’s people alright mate do you want to have a coffee like you know and have a little banter with them and like tap them on the shoulder and it’s so stressful for me. Because other people don’t realise that he’s you know he’s got Alzheimer’s so they just think he’s not obeying the rules.”Female carer (daughter), Baseline ID24.*

### THEME 3: putting oneself in danger

As a result of often mixed and limited comprehension of the various public health restrictions, some carers reported how the PLWD they were caring for was putting themselves, unbeknownst to them, in danger. This is for example evidenced by some PLWD entering a supermarket without a face covering and unaware of social distancing rules, whilst other PLWD were reported to not remember the need for social distancing and therefore were more likely to be in close proximity to others if outside.

“*So we got to the till and there was all this thing, I went no mum you’ve got to wait on the arrow ‘til you’re called or you know ‘til the man goes and then we can go and she just said well I think that’s utterly ridiculous that shop will end up closing down it will do no business and I said mum this is what all the all the shops and all supermarkets, well the supermarkets have all been like this for the last 3 months mum. So I took her home and the next day when I rang her she said oh I went to that new Morrison’s yesterday it’s not going to do any good at all, there were stupid rules in there about keeping like it wasn’t me who’d taken her.”Female carer (daughter), Follow-up ID30*

*“I couldn’t pull [PLWD] away from somebody because he can’t move quick enough and if that person didn’t if that person who as I say was approaching us didn’t move. We wouldn’t be able to social distance because we can’t make [PLWD] do it. Because he doesn’t understand what I’m doing, if anything he would he would levitate towards the person.”Female carer (spouse), Follow-up ID34*

*“at first it was hard because the weather wasn’t really great and my Dad didn’t understand that you can’t get close to people and he’s very you know like pats people on the back and shakes their hand. Every time we’d go for a walk even if it’s just the canal. He doesn’t understand you can’t do that.”Female carer (daughter), Baseline ID24*

*“he doesn’t understand the thing about like keeping the distance when you're out and that”Female carer (spouse), Baseline ID41.*

This caused additional stress to carers, as they were concerned about whether PLWD would adhere to restrictions and thus the need to constantly remind PLWD about the restrictions and why these were in place.

### THEME 4: adherence to restrictions in wider society

Some carers shared stories of how they struggled adhering to the public health measures when out in public with the PLWD. Going shopping was the main example, as often shop assistants would not allow more than one person in at any one time, resulting in the PLWD being left outside in some situations. This caused severe distress to carers, with some unable, or unwilling, to enter shops in the first place due to their previous upsetting experiences. However, many carers equally noted no specific difficulties on a societal level, so that it is important to highlight that these difficulties were not experienced by all.

*“[my wife] understands what covid’s about but she doesn’t fully appreciate all the consequences and if we want to go shopping they say it’s only one person, I can’t leave my wife on her own. Some stores have been really good. I mean there's a guy in [supermarket], there's a guy in [supermarket] and they just go ‘yeah don't have to explain yourself’, you're fine go in. Other people are saying well ‘no, one will have to sit in the car’, That’s been difficult at times because we've had to walk away from some shops because I just won’t leave my wife on her own because she will get distressed and it’s not fair, she doesn't deserve it”Male carer (spouse), Baseline ID47*

*“as soon as we walked in this absolute Rottweiler behind the counter shouted only 1 in the shop. So I said excuse me I said I have to be with my mum she is not allowed to shop on her own, she’s partially sighted and she has dementia. So she just glared at me so I said to my mum what do you want and she said oh have they got any Eccles cakes and this woman shouted they’re at the end right there on display. We went through the whole thing and when mum had decided exactly what she wanted I said mum just go and stand outside by the door please while I pay and this assistant then had the cheek to shout to me are you going to leave her out there..”Female carer (daughter), Follow-up ID30.*

### THEME 5: unchanged perceptions

There appear to have been no changes in understanding of public health restrictions between baseline and follow-up interviews. Specifically, restrictions involve the imposed lockdown and government ruling to stay indoors wherever possible, and to leave the house a maximum of once a day for exercise or essentials (food, health care). Carers reported their PLWD to be consistently confused about why certain behaviours and measures were in place, such as only seeing a family member through a window for a care home resident.

*“She doesn’t understand if I’m outside why I’m not going physically into the building.”Female carer (daughter), Follow-up ID35 – care home resident*

*“to a point you have to just keep reminding him about the social distancing he will always put his mask on if we go in the shops. He lives in supported living, although it’s a private flat and obviously those that need the domiciliary care there's people going in doing that. He doesn’t. Because he's got us, he doesn’t need that at that point, at this moment in time. But again he washes his hands; he's all aware of all that sort of thing. But he always just comes, I go shopping with him now. I don't know whether he’d feel confident enough going on his own at the moment.”Female carer (daughter), Follow-up ID40.*

## Discussion

This is the first study to report on the level of understanding of and adherence to COVID-19 public health measures in PLWD. Comprehension, and as a result adherence, was mostly very poor in PLWD, and linked to occasional behavioural problems, including agitation and frustration. Lack of comprehension also resulted in some PLWD putting themselves in dangerous situations without adhering to public health measures, with Society not always supportive and adaptive to the needs to PLWD in light of the restrictions.

Most PLWD, as reported by their carers, failed to comprehend most or all public health measures, and had to be reminded several times a day as to why they were not allowed to continue going to support groups or going for walks outside. As PLWD who participated in the study were aware of the restrictions and adhered to them, it is likely that PLWD who took part in our study were in the earlier stages of the condition as they also had capacity to consent. This suggests that the more advanced the dementia, the more difficult it is for PLWD to retain the new, and frequently changing, information about public health measures, in line with their deterioration of short-term memory abilities [3]. Thus, PLWD in the more advanced stages are likely at greater risk to put themselves in dangerous situations, such as entering supermarkets without face coverings and not adhering to hygiene and social distancing rules, due to their lack of understanding and ability to process new information. This suggests an urgent public health need to protect PLWD better.

It is important to place the experiences and understandings of the restrictions into context. At the point of data collection, the UK was in lockdown 1 from March to late June, with restrictions eased late June/ early July, when follow-up interviews were collected. Since then, the UK has seen two further lockdowns (lockdown 2 in November 2020; lockdown 3 since 31st of December 2020), as well as nation-specific restrictions. England, Wales, Scotland, and Northern Ireland are deciding their own levels and types of public health restrictions, and in England, a tier system has been introduced as of summer 2020. Each Tier (outside of lockdown times) has been rated based on the levels of infections, with most recently prior to lockdown 3 a 4-Tier system having been in place. Tier 4 restrictions were effectively like a lockdown scenario, with all non-essential retail and food and drink outlets closed, and people only recommended to leave the house for exercise or to access essential services (i.e. supermarkets or health care services). This also involved not seeing anyone indoors or outdoors except 1 person outdoors from a different household or someone from within a support bubble. Even for people without dementia these rapid and constant changes, although necessary, have become difficult to follow at times. Thus, the difficulties PLWD have experienced at the time of data collection are likely to be further exacerbated since with restrictions much more varied than previously.

PLWD are not the only ones affected by these restrictions however. Due to the lack of understanding of public health measures, PLWD can experience greater behavioural difficulties, such as agitation, which are already common in dementia in general [12]. Thus, it appears that the public health measures in place have further increased behavioural issues, which in turn can be difficult to cope with for carers. Caring for someone with dementia can be stressful and very demanding in itself [13], with COVID-19 having placed additional levels of burden onto unpaid carers. Carers are vital in supporting PLWD living well and independently at home, but this also raises the issue of carers needing to be considered as people requiring support in their own right, in line with the Care Act [14].

**Fig. 1:**
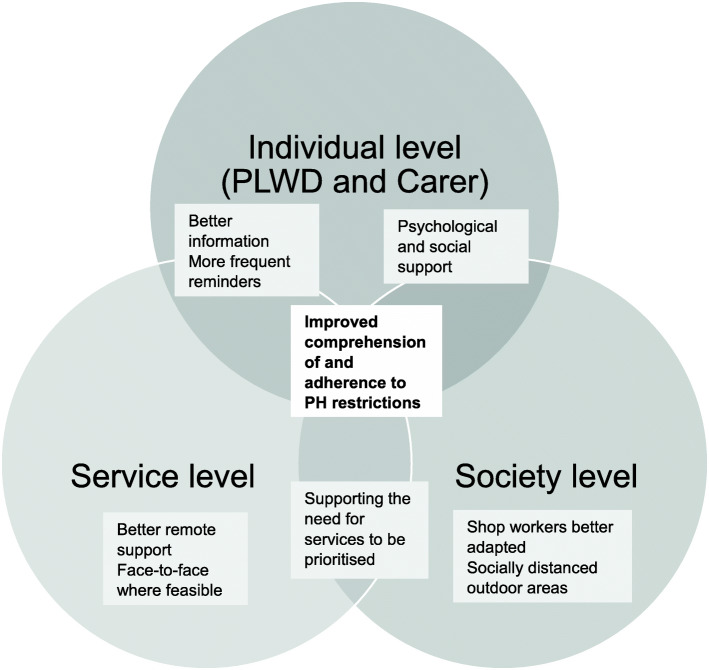
Public health recommendations and tailoring for dementia. Legend. COVID-19 public health restrictions need to be tailored on three levels to benefit the person living with dementia and carers: (1) Individual level; (2) Service level; (3) Society level. Tailoring recommendations and supporting the implementation of recommendations on a service and society level underpin a more effective adherence to public health restrictions on an individual level. Grey highlighted text boxes highlight example recommendations on each of the three levels. PH – Public health; PLWD – People living with dementia

We therefore propose COVID-19 public health measures to be tailored and implemented better on three different levels to benefit PLWD and carers: On an individual, service, and society level. Figure 1 illustrates these levels of recommendations and how these are interlinked.

On an *individual level*, both the PLWD and the carer need to be addressed separately. Information on public health measures needs to be simpler and clearer for PLWD to understand, and not only in the form of written guidance, but other means including videos for example. As PLWD struggle retaining information depending on the severity of the condition, PLWD require frequent reminders. Thus, clearer guidance via different channels could potentially be one way to reduce some carer burden, with carers less likely required to repeat the restrictions to the PLWD. Carers need to be recognised as persons in their own right, not as an attachment to a PLWD. Due to the increased burden of reminding PLWD about the restrictions and ensuring they adhere to these, better psychological and social service support is required for carers to support them in supporting the PLWD well. This can involve psychotherapy, Cognitive Behavioural Therapy, or any other means of therapy for example, and is fully dependant on the needs of the carer.

On a *service level*, services need to be adapted better to providing remote support. The pandemic is going to stay for the foreseeable future, despite vaccinations at different rates in each country and considering emerging variants of the virus, with likely easing and imposing of tighter restrictions again. Services therefore need to be delivered remotely as much as possible, by for example transferring dancing classes to creating videos for PLWD and carers to watch and enjoy together as opposed to attending classes in pre-pandemic times. It is important to recognise the barriers to remote support for many PLWD however, either due to their dementia subtype and vision difficulties, or due to high rates of digital illiteracy in older adults, or financial reasons and being unable to afford a computer and internet [10, 15]. One way to slightly overcome the barrier of digital illiteracy in many older adults may be to provide services, where possible, over the phone, and to provide assistance in using the internet and interventions to improve digital skills. As evidenced, remote support cannot replace face-to-face services, and many PLWD and carers do want direct social contact with their peers [10]. Given that many PLWD are aged 70+ and might have underlying health conditions, thus classifying them as vulnerable to the virus, it may be impractical at high-risk periods of the pandemic to recommend face-to-face services in a safe fashion (i.e. socially distanced, face coverings). To ensure that services can continue to exist throughout the pandemic and provide full face-to-face support again afterwards, it is important for the government to support the often small businesses providing such services.

On a *societal level*, we recommend that staff in shops and services are more aware of ‘hidden disabilities’, such as dementia or learning disabilities, where people with the condition are not always able to understand restrictions, adhere to them, or be left on their own without their carer. One way is by increasing the rollout of ‘Dementia Friends’ for example. This has already been picked up within discussions of the policy of wearing face masks and disability laws in the US for example [16], and highlights an urgent need for society to support and accommodate PLWD, amongst others, better in staying safe during the pandemic. Another way for society to adapt restrictions and enable PLWD to still engage in an active social and physical life is for example for outdoor spaces such as parks to create socially-distanced areas for PLWD and other vulnerable groups.

These are only some recommendations on how public health restrictions can be adapted and tailored to support the needs of both PLWD and carers, and aligns with a recent report British Psychological Report on how to support vulnerable people, including those living with dementia, coming out of ‘shielding’ [17]. In light of the severe impact of public health measures on the mental and physical lives of PLWD and carers, these recommendations can be broadened by having in-depth co-production workshops with service providers, local councils, clinicians, as well as those affected by dementia.

## Conclusions

PLWD are severely struggling to comprehend COVID-19 public health restrictions and retaining information about these. As a result, carers are struggling where these are available, as they have to repeatedly inform the PLWD about these restrictions, and safeguard them to adhere to the restrictions. With the current second wave of COVID-19 cases and a new round of stricter public health restrictions in place, we recommend public health measures to be tailored and implemented on three specific levels to support PLWD and carers: on an individual, service, and society level. These recommendations will not only be suitable to PLWD, but also other groups with cognitive deficits or hidden disabilities, such as learning disabilities and autism. Considering that the pandemic and associated public health restrictions are going to stay around for the foreseeable future, it is vital that we take learning from the first wave of the pandemic and adapt and tailor measures to support the well-being of those with dementia and other cognitive deficits, and their carers.

## Supplementary Information

**Additional file 1.**

## Data Availability

Transcripts are available upon reasonable request from the lead author. This research is supported by a grant awarded to the authors by the University of Liverpool COVID-19 Strategic Research Fund in 2020. This is also independent research funded by the National Institute for Health Research Applied Research Collaboration North West Coast (ARC NWC). The views expressed in this publication are those of the author(s) and not necessarily those of the National Institute for Health Research or the Department of Health and Social Care.

## Abbreviation

PLWD: People living with dementia
