# Supplementary material for: The potential dangers of not understanding COVID-19 public health restrictions in dementia: “It’s a groundhog day – every single day she does not understand why she can’t go out for a walk”
Source: BMC Public Health. 2021 Apr 20;21:762. doi: 10.1186/s12889-021-10815-8 (PMC8057664; doi:10.1186/s12889-021-10815-8)
Supplement: Supplementary file 1 — Additional file 1. [file 12889_2021_10815_MOESM1_ESM.docx]

**Appendix 1. Topic Guide**

Q1. Has your situation changed since we last spoke?

If you are caring for someone with dementia, has your caring situation changed?

If you are living with dementia, have there been any significant changes in your life?

Q2. What have been your experiences with social support services since the lockdown?

Have services adapted better in recent weeks?

If so, do these adaptations meet your needs?

Q3. What are your experiences with remote support, whether digital or telephone?

If you do receive remote support, does it benefit you?

If you do receive remote support, what are your experiences using digital technology?

Q4. Are you accessing any form of face-to-face support, and if so, what?

Q5. Reflecting back on when support suddenly changed back in March, how are you coping now? Do you feel you have all the support you need?

Q6. Is there anything else you feel is important to mention in terms of how COVID-19 has affected your ability to receive social support which we have not covered yet.
